# How the corona pandemic affects the global fight against tuberculosis and how to react

**DOI:** 10.3389/fcimb.2023.1165160

**Published:** 2023-04-04

**Authors:** Timo Ulrichs

**Affiliations:** ^1^ Institute for Research in International Assistance, Akkon University for Human Sciences, Berlin, Germany; ^2^ Koch-Mechnikov Forum, Berlin, Germany

**Keywords:** tuberculosis, COVID-19, global health, GFATM, pandemic

## Abstract

The emergence of the acute pandemic by SARS-CoV-2 is a setback for the fight against chronic pandemics like tuberculosis (TB), malaria, and HIV/AIDS. In fact, after more than a decade of decreasing fatality numbers, 2020 saw a re-increase in the number of people dying from TB. After COVID-19, TB was the infectious disease with the second-highest fatality rate caused by a single pathogen, with 1.6 million deaths in 2021. It is expected by the WHO that the pandemic years to come and even after the pandemic will continue this trend. More efforts are needed to support TB control structures as an integral part of the strengthening measures of the general health care system.

## 1 Epidemiology

Apart from the increased death toll, other epidemiological indicators clearly illustrate the setback in the global and regional endeavors to contain the spread of *Mycobacterium tuberculosis*: case notification rates for TB decreased in most of the high-burden countries, expressing a tremendous underreporting due to the fact that public health facilities were and still are overwhelmed by the workload caused by the corona pandemic (WHO, 2022). Human resources, laboratory, and clinical capacities were re-directed toward diagnosis and containment of SARS-CoV-2 rather than identifying new TB patients and providing correct anti-TB treatment. Although many countries implemented strict hygienic measures like social distancing, wearing face masks, and reducing individual contacts, resulting in a decrease of aerosol-driven infections of all air-borne pathogens, tuberculosis spread has not been decreased by these measures significantly, other than seasonal influenza, which virtually did not take place in many European countries in the autumn/winter season 2020/2021 (Stamm et al., 2021). Underreporting of TB cases leads to not only false-negative incidences in high-burden countries but also millions of undetected TB patients who cannot be integrated into the TB control programs. Their prognoses, of course, are worse than those of outpatients or hospital-admitted TB patients. The WHO estimates that the number of undetected TB cases increased from 2.9 million in 2019 to 4.1 million in 2020, with a tendency to continuously increase in 2021 and also 2022 (WHO, 2022). In the early phase of the pandemic, TB control even in the WHO European Region has been neglected, resulting in decreasing TB notification (Dara et al., 2021).

In addition to the re-direction of resources from TB to COVID-19, lockdown measures in many countries led to a shutdown of TB dispensaries and diagnostic centers, resulting in a situation of insecurities for TB patients undergoing an anti-TB treatment regimen with an increased risk of incomplete therapy adherence. This could have furthered the emergence of additional cases of multidrug-resistant (MDR) cases (including extensive MDR (XDR)), especially in countries with a high prevalence of already existing multidrug-resistant strains of *M. tuberculosis.* Even a delay in diagnosing new TB cases due to anti-corona measures can result in the progress of the clinical disease, lung tissue damage, and exacerbation of highly replicating *M. tuberculosis* in infected and diseased individuals. Exacerbating TB disease patterns will decrease treatment options and the overall prognosis (Di Gennaro et al., 2021; McQuaid et al., 2021).

If we look closer at the overall epidemiological situation according to the current WHO Global TB Report (WHO, 2022), the most obvious outer sign of the corona pandemic-driven setback of TB containment is the comparison of the current epidemiological data with goals of the planned decrease in TB numbers within the sustainable development goals (SDGs, End TB Strategy; 4, 7). In SDG 3, the global TB incidence should decrease by 80% and the TB fatality rate by 90% from 2015 to 2030. A midterm evaluation in 2020 revealed that the actual decrease in the incidence rate is at 11% instead of the planned 20%, and the decrease in fatality is at 9.2% instead of the planned 35%. However, in contrast to the global development, the WHO European Region registered a stronger decrease (incidences by 25% and fatality rate by 26%, mainly due to increased efforts and success of TB control in the Russian Federation) before the corona pandemic (WHO European Office, 2021). Not reaching the mid-term goals is not just an epidemiological issue. All not-prevented TB deaths contribute to increasing expenses, measured in direct and indirect costs of TB disease (Silva et al., 2021). In fact, the corona pandemic is responsible for increased expenses in TB containment, while the investments into TB control (diagnostics, therapy, and prevention) decreased from $5.8 billion in 2019 to $5.3 billion in 2020—this is less than half of the amount needed to reach the SDG and End TB Strategy goals, according to the calculations of the WHO (Satyanarayana et al., 2020; Stamm et al., 2021; WHO, 2022).

## 2 Immunology

In the first studies in TB high-burden countries, it has been revealed that COVID-19 mortality is two to three times higher in TB patients compared to non-TB patients suffering from COVID-19 (Di Gennaro et al., 2021). The question of whether the two pathogens, SARS-CoV-2 and *M. tuberculosis*, influence each other in furthering disease onset or clinical courses and in delaying or disturbing the specific immune response is still unclear. However, there are hints that SARS-CoV-2 infection interacts with TB diagnostics and comorbidity of COVID-19 and that TB presents with altered immunological responses in both diseases compared to single infections/diseases (Shah et al., 2022). Also, it seems that T-cell responses might influence each other, resulting in decreased defense abilities against TB or HIV/AIDS in COVID-19 patients (Riou et al., 2021), as well as vice versa (Petronea, 2021). Insights into the local host–pathogen interactions in lung tissue involving both pathogens and their specific lung pathology are still missing or have conflicting outcomes (Mousquer et al., 2021).

## 3 Lessons learned for TB control after the corona pandemic

Acute global health emergencies can alter the ongoing programs in fighting infectious diseases. TB, a global health emergency itself since 1993 (WHO, 2022), is a very complex health threat, as *M. tuberculosis* is an intracellular pathogen, and the immune response requires a well-orchestrated activation of different parts of the immune system. Immunity against the virus SARS-CoV-2 is much more straightforward but also requires the involvement of specific antibody production and T-cell responses and memory. The interactions between the two infectious diseases are not yet fully understood, but it is quite clear that a co-infection might aggravate the clinical courses of both diseases (Shah et al., 2022). Thus, preventive measures should aim at avoiding viral spread among TB patients in high-burden countries as one of the public health priorities or allow only slow progress of the transmission. To enable healthcare systems in poor countries to tackle both diseases, financial and structural support is needed soon (Dara et al., 2021; WHO, 2022). Unfortunately, poor countries have the least coverage for anti-corona vaccination. To mitigate the long-term effects of the pandemic, the COVAX initiative (i.e., delivery of anti-corona vaccines to countries in need) should be intensified, even in times when herd immunity against the coronavirus has been reached in rich countries. In addition, the Global Fund to Fight AIDS, Tuberculosis and Malaria (GFATM) responded to this additional challenge by launching a special program on COVID-19 containment (Friends of the global fund, 2022). This could initiate a change of paradigms, namely, that global programs like the one to end TB (within the SDG framework (Satyanarayana et al., 2020) should be organized with higher resilience toward additional challenges like the corona pandemic. Furthermore, funding programs to support the fight against single infectious diseases like the programs of the GFATM should be re-arranged in a way that more comprehensive support of healthcare structures in high-burden countries rather than having vertical TB- or HIV-focused programs could enable health institutions to maintain their control programs and at the same time face the new challenge by the emerging viral pandemic. Finally, the corona pandemic clearly demonstrated the need to better link basic immunological research to applied research in public health concepts of pathogen containment and the necessity to increase funding for both. Multilateral and multicenter approaches could contribute to this link, e.g., combining basic research on avian influenza and at the same time investigating potential pandemic preparedness planning for an influenza pandemic, based on immunological insights of host–pathogen interactions. Research approaches in well-established settings like tuberculosis or the influenza field can serve as role models for investigating other zoonotic diseases with pandemic potential and for translating the results into better pandemic preparedness planning.

## Data availability statement

The original contributions presented in the study are included in the article/supplementary material. Further inquiries can be directed to the corresponding author.

## Author contributions

The author confirms being the sole contributor of this work and has approved it for publication.
